# Age patterns of intra‐pair DNA methylation discordance in twins: Sex difference in epigenomic instability and implication on survival

**DOI:** 10.1111/acel.13460

**Published:** 2021-08-24

**Authors:** Qihua Tan, Shuxia Li, Mette Sørensen, Marianne Nygaard, Jonas Mengel‐From, Kaare Christensen

**Affiliations:** ^1^ Epidemiology, Biostatistics and Biodemography Department of Public Health University of Southern Denmark Odense Denmark; ^2^ Unit of Human Genetics Department of Clinical Research University of Southern Denmark Odense Denmark

**Keywords:** aging, DNA methylation, epigenomic instability, mortality, twins

## Abstract

Aging is a biological process linked to specific patterns and changes in the epigenome. We hypothesize that age‐related variation in the DNA methylome could reflect cumulative environmental modulation to the epigenome which could impact epigenomic instability and survival differentially by sex. To test the hypothesis, we performed sex‐stratified epigenome‐wide association studies on age‐related intra‐pair DNA methylation discordance in 492 twins aged 56–80 years. We identified 3084 CpGs showing increased methylation variability with age (FDR < 0.05, 7 CpGs with *p* < 1e‐07) in male twins but no significant site found in female twins. The results were replicated in an independent cohort of 292 twins aged 30–74 years with 37% of the discovery CpGs successfully replicated in male twins. Functional annotation showed that genes linked to the identified CpGs were significantly enriched in signaling pathways, neurological functions, extracellular matrix assembly, and cancer. We further explored the implication of discovery CpGs on individual survival in an old cohort of 224 twins (220 deceased). In total, 264 CpGs displayed significant association with risk of death in male twins. In female twins, 175 of the male discovery CpGs also showed non‐random correlation with mortality. Intra‐pair comparison showed that majority of the discovery CpGs have higher methylation in the longer‐lived twins suggesting that loss of DNA methylation during aging contributes to increased risk of death which is more pronounced in male twins. In conclusion, age‐related epigenomic instability in the DNA methylome is more evident in males than in females and could impact individual survival and contribute to sex difference in human lifespan.

## Graphical abstract

Significant increase in DNA methylation variability with age is observed in males but not in females. Some of the CpG sites showing high age‐related variability are associated with mortality with reduced risk of death in samples maintaining high methylation levels at the high‐variability sites.

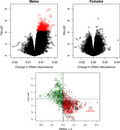

## 1 INTRODUCTION

The epigenetic regulation of gene activity during the aging process has been intensively investigated during the past decade taking advantage of the rapid development in high‐throughput techniques for DNA methylation (DNAm) analysis at genome scale. Multiple epigenome‐wide association studies (EWAS) have been performed to examine the progressive changes in DNAm accompanying aging. Large numbers of differentially regulated CpG sites have been reported which suggest that epigenetic changes have a strong influence on the aging processes (Li et al., 2017; Moore et al., 2015; Reynolds et al., 2020; Tan et al., 2016). Efforts have been made to find sex‐specific patterns of age‐related changes in the level of DNAm but with no obvious difference between the two sexes on autosomal chromosomes (Jansen et al., 2019; Tan et al., 2016), although a recent analysis reported sex‐dependent methylation patterns by age on the X‐chromosome (Li et al., 2020).

The identified age‐dependent changes in DNAm are directional with CpGs showing increased and decreased methylation (hyper‐ and hypomethylation, respectively) patterns occupying specific genomic regions enriched for different biological functions (Li et al., 2017). The reported mean levels of hyper‐ or hypomethylation are non‐stochastic with a predominant pattern of hypomethylation observed in the older subjects. For example, Li et al., (2017) reported a significantly higher proportion of hypomethylation (61%) with age in two large Lothian Birth Cohorts (Deary et al.,^2012^) and Johansson et al. (2013) reported an even higher proportion of hypomethylation (64%), re‐affirming the notion that the aging‐associated epigenetic modification is characterized by gradual and extensive demethylation of the DNA methylome (Zampieri et al., 2015).

Instead of focusing on the mean levels of age‐dependent change in DNAm, examining the variability of DNAm across individuals could better reflect individual aberration of DNAm which introduces genomic instability that potentially affects lifespan and healthspan in a sex‐specific manner (Fischer & Riddle, 2018). As individual methylation levels are subject to changes due to genetic and environmental factors, comparison across individuals is a challenging task due to individual heterogeneity and differential exposures. Although individual difference in genetic make‐ups can be controlled by taking repeated measurements on DNAm over ages in a longitudinal design, it is time‐consuming and expensive. Twin pairs, especially monozygotic (MZ) twin pairs who share the same genetic make‐ups, are ideal samples to fulfill the need (Tan et al., 2013, Tan, 2019). Intra‐pair DNAm difference in MZ twins can be compared across twin pairs of different ages to infer age‐related change in DNAm variability as twins in a pair are of the same age. This can be done in male and female MZ twins separately to examine sex differences in age‐related DNAm variability as MZ twin pairs are of the same sex. In fact, (Fraga et al.^2005^) already reported increased discordance in DNAm in a 50‐year‐old MZ pair when compared with that in a 3‐year‐old MZ pair. At the Danish Twin Registry (Pedersen et al., 2019), we have over the years accumulated genome‐wide DNAm data on multiple cohorts of twins enabling us to unprecedentedly analyze site‐specific age‐dependent intra‐pair DNAm variation in twins. The multiple twin cohorts allowed us to perform sex‐stratified discovery and replication analyses on independent samples. The identified significant sites were further characterized for their impact on mortality in cohorts of elderly twins in combination with bioinformatics analysis for functional interpretations.

## 2 MATERIALS AND METHODS

### 2.1 Study population

#### 2.1.1 The discovery cohort

The discovery stage analysis was performed on the Middle‐aged Danish Twins (MADT) consisting of twin pairs born between 1931 and 1952 available from the Danish Twin Registry (Gaist et al., 2000). DNA methylation analysis was performed on 492 blood samples (243 MZ and 3 same‐sex dizygotic or DZ pairs, 133 male and 113 female pairs) of subjects aged from 56 to 80 years with a median age of 66 years (Table 1), using the Infinium Human Methylation 450 K array (Illumina, San Diego, California, United States). Detailed descriptions of the cohort and laboratory analysis of DNAm can be found elsewhere (Starnawska et al., 2019).

**TABLE 1:** Basic description of all cohorts

Cohort	Sample size	Age at blood sampling		Twin pair			Age at death		
		Range	Median	MZ	DZ	Total	n	Range	Median
Discovery, MADT									
Male	266	57–80	66	132	1	133	40	63–87	77
Female	226	56–79	66	111	2	113	19	66–88	79
Total	492	56–80	66	243	3	246	59	63–88	78
Replication, BWD									
Male	152	30–74	37	76		76			
Female	140	30–74	57	70		70			
Total	292	30–74	57	146		146			
Mortality, LSADT									
Male	72	73–88	78	36		36	72	76–97	86
Female	152	73–90	79	67	9	76	148	73–102	89
Total	224	73–90	78	103	9	112	220	73–102	89

#### 2.1.2 The replication cohort

The replication cohort consisted of 292 samples (152 males and 140 females) from 146 pairs of MZ twins aged 30–74 years with a median age of 57 years (Table 1). DNAm data were collected using the same 450K array as in the discovery cohort and were originally generated in connection with an EWAS on birth‐weight discordance (BWD) reporting no significant findings (Tan et al., 2014). Both raw and processed DNA methylation data have been deposited to the NCBI GEO database http://www.ncbi.nlm.nih.gov/geo/ under accession number GSE61496.

#### 2.1.3 Cohorts for mortality analysis

The samples used for the mortality analysis came from the Longitudinal Study of Aging Danish Twins (LSADT). The LSADT study, based on the Danish Twin Registry, is a cohort sequential study of 224 elderly Danish twins (103 MZ and 9 same‐sex DZ twin pairs, 72 males and 152 females) (Table 1). LSADT began in 1995 with an assessment of all members of like‐sex twin pairs born in Denmark before 1920. Blood samples were drawn during home visits in 1997 (median age 79, range: 73–91) from which DNA was isolated and DNA methylation measured using the same 450K array as in the discovery and replication cohorts (Tan et al., 2016). Dates of births and deaths were obtained from the Danish Civil Registration System in September 2020, at which point in time 220 twins had died (median age at death 89 years, range: 73–102).

In the MADT cohort, 59 twins died at a median age of 78 years (range: 63–88) (Table 1). The MADT survival data were used for replicating results of survival analysis on LSADT cohort.

#### 2.1.4 Ethical approvals

Permissions to collect blood samples and the usage of register‐based information were granted by Regional Committees on Health Research Ethics for Southern Denmark (S‐VF‐19980072), with specific permissions for MADT and LSADT (S‐VF‐20040241) and for BWD (S‐20090033) cohorts. All studies were conducted in accordance with the Helsinki II declaration.

### 2.2 DNAm data preprocessing

For each dataset of Danish twins, normalization on the measured DNA methylation level was performed by the functional normalization (Fortin et al., 2014) implemented in the R package *minfi*. Probes with a detection p value (a measure of an individual probe's performance) >0.01 were treated as missing. CpG sites with more than 5% missing values were removed from the study. After quality control and filtering, a total of 292376 CpGs remained. At each CpG site, DNAm level was summarized by calculating a “beta” value defined by the Illumina's formula as β = M/(M + U + 100), where M and U are methylated (M) and unmethylated (U) signal intensities measured at the CpG site. Before statistical analysis, the methylation β values were transformed into M values using the logit transformation with M = log_2_(β/(1−β)) for better statistical properties in fitting regression models.

### 2.3 Adjusting for blood cell composition

Since DNA methylation was measured in whole blood comprising multiple cell types, cellular heterogeneity among twin samples can be an important factor influencing DNAm due to cell specificity of DNA methylation. To control for the effect of cell type composition, the proportions of major leukocyte cell types were estimated using the Houseman method (Houseman et al., 2012) implemented in the R package *minfi*. Based on the DNA methylation data, the method estimated blood cell composition in each individual twin for 6 blood cell types: CD8T, CD4T, natural killer cells, B cells, monocytes, and granulocytes. In the data analysis, we first regressed DNAm M value on the estimated cell type proportions and then kept the residual for each CpG for downstream statistical analysis.

### 2.4 Statistical analysis

#### 2.4.1 Association of age with DNAm variability

The age‐dependent DNAm variability is assessed by modeling the difference in DNAm within a MZ twin pair (absolute value) as a function of age in a simple linear regression model as

$$|\text{DNAm(twin1) - DNAm(twin2)| = |}\Delta \text{DNAm| =}\;\beta_{0}+\beta_{1}\text{age}$$

By testing the null hypothesis Ho: β_1_ = 0, the age‐related change in DNAm variability can be detected if β_1_ is significantly different from 0, with β_1_ > 0 or β_1_ < 0 indicating increased or decreased variability in DNAm with age (two‐sided test). In order to assess sex differences in DNAm variability with age, we fitted the above model to male and female twin pairs separately. In the discovery stage, statistical significance of the CpGs was determined by calculating the false discovery rate (FDR) (Benjamini & Hochberg, 1995) and CpGs with FDR<0.05 were defined as genome‐wide significant. Top significant CpGs were defined by Bonferroni corrected *p* values depending on the number CpGs tested.

#### 2.4.2 Survival analysis

For CpG sites detected as showing significant age‐dependent variability, we further assessed their association with risk of death in an older twin cohort with death records. To do that, we fitted the Cox proportional hazard model with DNAm measurements as an explanatory variable and defined twin pairing as clusters to account for twin correlation in survival time while adjusting for age at blood sampling.

#### 2.4.3 Over‐representation analysis

Over‐representation analysis (ORA) is used to assess if a submitted list of significant CpGs from a replication cohort contains CpGs overlapping with significant CpGs from the discovery cohort more than would be expected by chance by calculating the probability from a hypergeometric distribution, that is,

$$p\left(X\geq k\right)=1‐\sum_{r=0}^{k}\left(\begin{array}{c} m\\ r \end{array}\right)\left(\begin{array}{c} N‐m\\ n‐r \end{array}\right)/\left(\begin{array}{c} N\\ n \end{array}\right)$$

where *N* is the number of all CpGs on the 450k array, *m* is the number of significant CpGs found in the replication cohort, *n* is the number of significant discovery CpGs, and *k* is the number of overlapping CpGs.

### 2.5 Biological pathway analysis

To test if genes in one biological pathway are over‐represented by genes annotated to a list of significant CpGs, a gene‐set enrichment analysis (GSEA) was performed. By replacing *N* in the equation above with the number of all genes linked to the CpGs on the 450K array, *n* with a list of genes belonging to biological pathway in *N*, *m* with a list of genes linked to all significant CpGs, *k* with the number of overlapping genes between *n* and *m*, a hypergeometric probability can be calculated. GSEA was performed as ORA of canonical pathways at https://www.gsea‐msigdb.org/gsea/index.jsp. Annotation of CpGs was done using the Bioconductor package: IlluminaHumanMethylation450kanno.ilmn12.hg19.

## 3 RESULTS

### 3.1 Discovery EWAS on DNAm variability

**FIGURE 1:**
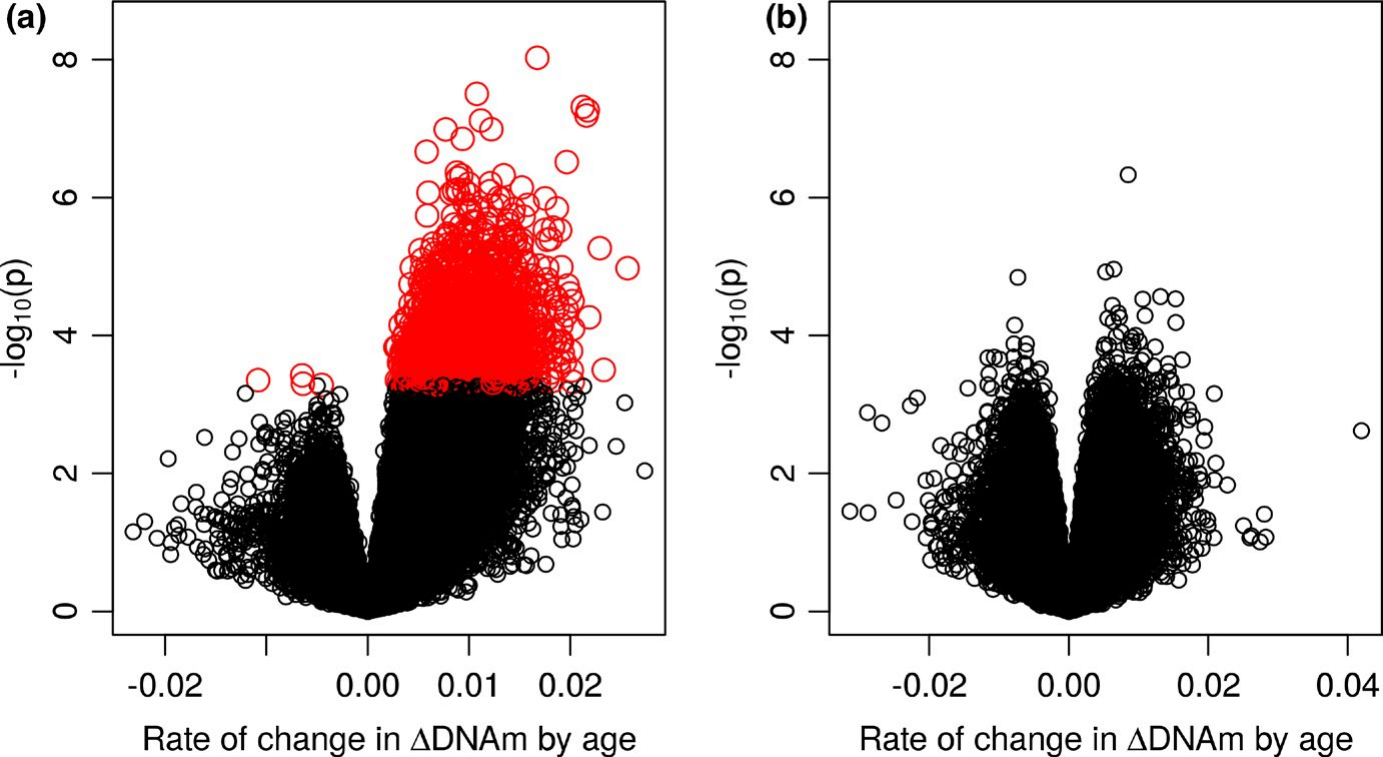
Volcano plot of discovery EWAS on MADT male (a) and female (b) twins, with negative log of the p value (base 10) for each CpG site on the y‐axis. The red dots are CpGs showing significant age‐related intra‐pair methylation discordance with FDR < 0.05

**FIGURE 2:**
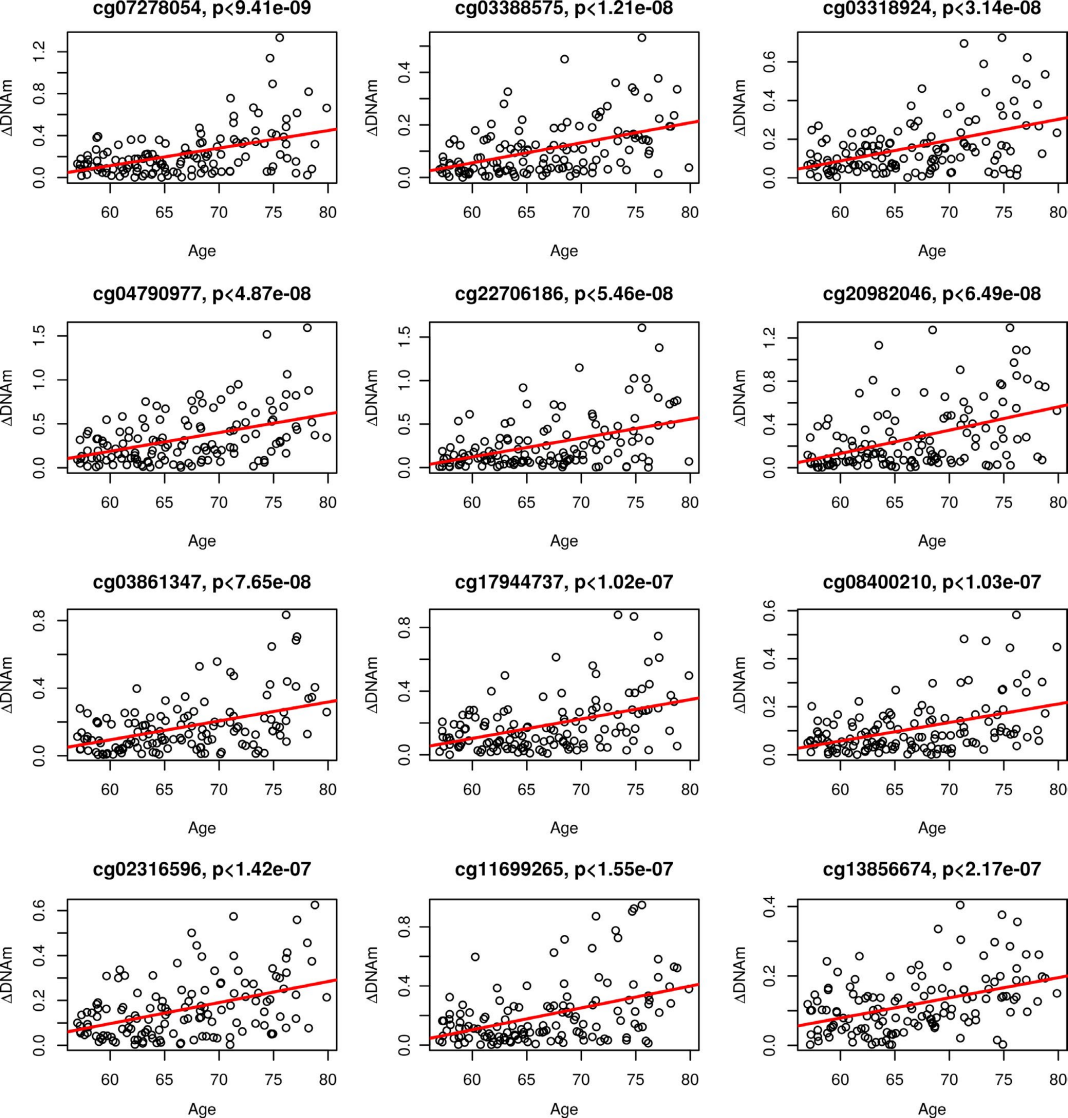
Scatter plots of intra‐pair DNAm discordance of each twin pair plotted against age of twin pair for top 12 CpGs significant in males with FDR ≤ 0.005

By performing EWAS of age‐related DNAm variability on male and female MADT samples separately, we detected 3089 CpGs showing significant increase (3084 CpGs) or decrease (5 CpGs) in intra‐pair DNAm discordance by increasing age in males with FDR < 0.05 (corresponding to *p* < 5.25e‐04) (see EWAS results in Table S1). In contrast, no significant CpGs were found in female samples (the lowest FDR detected 0.138, corresponding *p* value 4.69e‐07) as shown by the volcano plots in Figure 1. Using Bonferroni correction, we selected CpGs with *p* < 1e‐07 as top significant sites that meet both criteria. In males, we identified 7 CpGs with *p* < 1e‐07, which are linked to the *ELFN1*, *C1QL4*, *FAM19A1*, and *SULF2* genes, but no CpG from female twins (Table 2). In Figure S1, the p value of each CpG site is plotted against its base pair position in the Manhattan plots for males (S1a) and females (S1b) displaying much higher statistical significance in male than in female samples. The age‐related change in intra‐pair DNAm discordance is plotted in Figure 2 for the top 12 significant CpGs (*p* ≤ 2.17e‐07) in Table 2 with significant increase in DNAm variability displayed for all CpGs. In Figure S2, the distribution of the 3089 significant CpGs is shown over gene regions (S2a) and relative location to CpG islands (S2b). The distribution (red curve) is, overall, not different from that of all CpGs (blue curves) on the 450K array.

**TABLE 2:** Top significant discovery CpGs found in male twins (*p* < 1e‐06)

CpG IDs	Coef.	SE	*p* value	FDR	chr	Position	Relation to CpG island^a^	UCSC RefGene Name	RefGene Group
cg07278054	0.017	0.003	9.41E‐09	0.002	chr3	55523594	S_Shore		
cg03388575	0.008	0.001	1.21E‐08	0.002	chr7	1765158	OpenSea	ELFN1	5'UTR
cg03318924	0.011	0.002	3.14E‐08	0.003	chr12	49728150	N_Shore	C1QL4	Body
cg04790977	0.021	0.004	4.87E‐08	0.003	chr2	220717120	OpenSea		
cg22706186	0.022	0.004	5.46E‐08	0.003	chr3	68053371	N_Shelf	FAM19A1	TSS200
cg20982046	0.022	0.004	6.49E‐08	0.003	chr8	74282931	OpenSea		
cg03861347	0.011	0.002	7.65E‐08	0.003	chr20	46412713	N_Shore	SULF2	5'UTR
cg17944737	0.012	0.002	1.02E‐07	0.003	chr1	10995014	OpenSea		
cg08400210	0.008	0.001	1.03E‐07	0.003	chr6	89908154	OpenSea	GABRR1	Body
cg02316596	0.009	0.002	1.42E‐07	0.004	chr11	120434979	CpG Island		
cg11699265	0.015	0.003	1.55E‐07	0.004	chr7	98990265	CpG Island	ARPC1B	Body
cg13856674	0.006	0.001	2.17E‐07	0.005	chr15	93722590	OpenSea		
cg08373528	0.020	0.004	3.06E‐07	0.007	chr6	42672105	OpenSea	PRPH2	Body
cg18054745	0.013	0.002	3.88E‐07	0.008	chr11	1370793	OpenSea		
cg25925023	0.009	0.002	4.35E‐07	0.008	chr2	220299484	CpG Island	SPEG	TSS1500
cg00229368	0.013	0.003	4.78E‐07	0.008	chr3	184279621	CpG Island	EPHB3	1stExon; 5'UTR
cg11734329	0.009	0.002	4.79E‐07	0.008	chr19	7460671	S_Shore	ARHGEF18	5'UTR
cg12791555	0.009	0.002	5.22E‐07	0.009	chr15	75118714	OpenSea	CPLX3	TSS1500
cg14275340	0.012	0.002	6.23E‐07	0.009	chr14	58862450	N_Shore	TOMM20L	TSS200
cg17921439	0.010	0.002	6.33E‐07	0.009	chr10	128211324	OpenSea	C10orf90	TSS1500
cg22746182	0.015	0.003	7.18E‐07	0.009	chr8	41749867	N_Shelf	ANK1	Body
cg12683120	0.009	0.002	7.66E‐07	0.009	chr20	50182196	S_Shelf		
cg04467618	0.010	0.002	7.79E‐07	0.009	chr6	134210946	CpG Island	TCF21	1stExon
cg12856183	0.009	0.002	8.01E‐07	0.009	chr15	89953033	CpG Island		
cg00808170	0.012	0.002	8.13E‐07	0.009	chr5	140807787	CpG Island	PCDHG	Body
cg12011897	0.006	0.001	8.51E‐07	0.009	chr12	57119209	CpG Island	NACA	TSS200; 5'UTR; 1stExon
cg18158033	0.009	0.002	8.52E‐07	0.009	chr15	99975010	OpenSea		
cg23043544	0.008	0.002	8.67E‐07	0.009	chr7	2124092	OpenSea	MAD1L1	Body
cg03167699	0.010	0.002	8.96E‐07	0.009	chr11	6708820	S_Shelf		

^a^ CpG island: regions of greater than 500 bp that have guanine‐cytosine content of greater than 55%; Shore: regions 0–2 kb from CpG islands; shelves: regions 2–4 kb from CpG islands; open sea: regions of isolated CpG sites in the genome that do not have a specific designation. The up to 2 kb sequences, directly up‐ and downstream of CpG islands are called the northern and southern shore (N_Shore, S_Shore), respectively. The 2 kb sequences directly adjacent to the shores are called the northern and southern shelves (N_Shelf, S_Shelf).

### 3.2 Sensitivity analysis of discovery EWAS

In Table 1, the number of male twins is larger than the number of female twins in the MADT cohort (266 vs 226). To ensure that the pattern of sex difference in Figure 1 is not due to differences in sample size, we performed a sensitivity analysis by re‐doing the EWAS on DNAm variability in male twins assigning an equal sample size as for female twins (113 random pairs of male twins). The volcano plot in Figure S3 confirms the sex difference pattern seen in Figure 1, although with a reduced number of significant CpGs in males (306 CpG with FDR<0.05 corresponding to *p* < 5.42e‐06) due to the reduced sample size. In subsequent analysis, we stick to estimates from the full sample of male MADT twins with the most powerful setup possible.

### 3.3 Replication using an independent twin cohort

**FIGURE 3:**
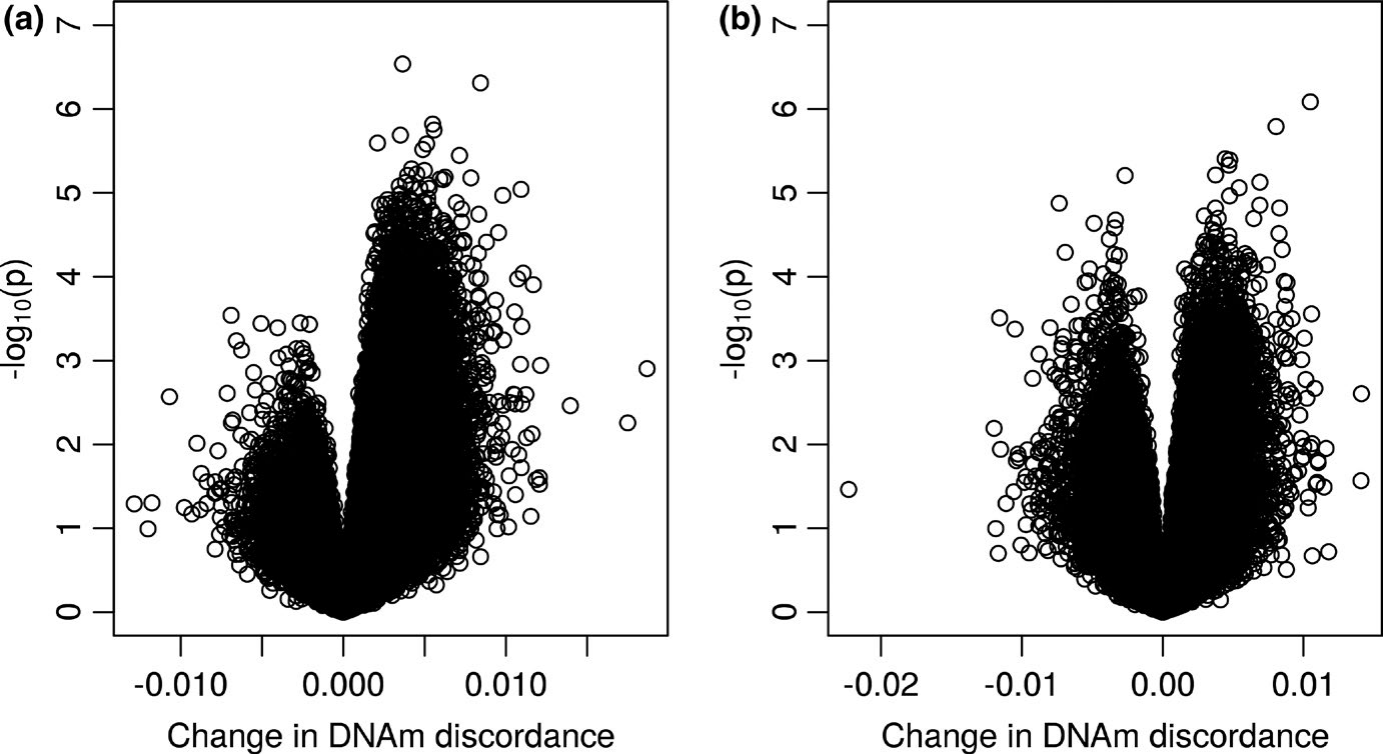
Volcano plot of replication EWAS on BWD male (a) and female (b) twins, with negative log of the p value (base 10) for each CpG site on the y‐axis

**FIGURE 4:**
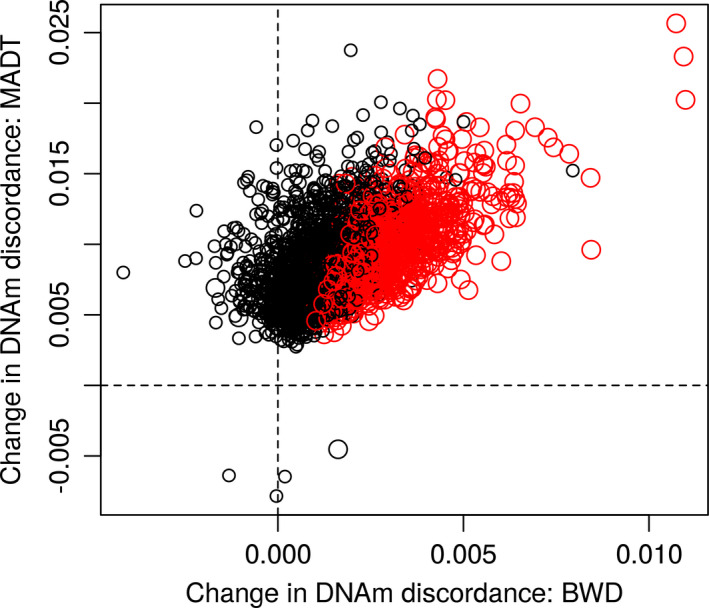
Rate of change in intra‐pair DNAm discordance in male MADT twins (*y*‐axis) plotted against that in male BWD twins (*x*‐axis) with red dots representing CpGs replicated with *p *< 0.05 in BWD twins account for 37% of discovery CpGs

For replication, the same model for discovery EWAS was applied to the BWD twins with two purposes, (1) replicating the sex‐specific patterns in Figure 1 and (2) replicating the 3089 discovery CpGs. In Figure 3, the replication volcano plots display more significant CpGs in male as compared with female samples. The pattern in Figure 3 is consistent with the discovery pattern in Figure 1 but with lower statistical significance due to a smaller sample size and different age spans. For the 3089 significant discovery CpGs, 2620 CpGs were matched to BWD data because of differences in which CpGs passed through QC, among which 975 were replicated with *p *< 0.05 (4 CpGs with *p* < 1e‐05, 2 CpGs with *p* < 1e‐6) and same direction of age‐related change (corresponding to a replication rate of 37%). A hypergeometric test showed very high statistical significance of the overlap with *p* < 1e‐16. Figure 4 plots the change in intra‐pair DNAm discordance by age for the 2620 discovery CpGs matched to BWD data against that for BWD. The replicated CpGs (red dots) exhibit high correlation on age‐related DNAm variability between the two independent cohorts. Among the top 7 discovery CpGs with *p *< 1e‐07, 5 were matched to the BWD data of which 3 were replicated with *p *< 0.05 and same direction of age‐related change.

### 3.4 Functional annotations

Based on the biological annotation in Table S2, we performed functional analysis of the significant discovery CpGs. The genes linked to significant sites were submitted to GSEA websites for over‐representation analysis of KEGG pathways. Twenty‐one gene sets (Table 3) were significantly enriched with FDR < 0.05 including pathways in cancer, ECM‐receptor interaction, neuroactive ligand‐receptor interaction, focal adhesion, Wnt signaling pathway, basal cell carcinoma, axon guidance, small cell lung cancer, MAPK signaling pathway, glioma, and melanoma.

**TABLE 3:** Over‐represented KEGG pathways by genes linked to significant CpGs (FDR < 0.05) in male twins

Gene sets	No. Genes (K)	No. Genes in Overlap (k)	*p*‐value	FDR *q*‐value
Pathways in cancer	325	31	3.61 e^−9^	6.72 e^−7^
ECM‐receptor interaction	84	14	8.34 e^−8^	7.75 e^−6^
Neuroactive ligand‐receptor interaction	272	24	8.46 e^−7^	4.24 e^−5^
Focal adhesion	199	20	9.12 e^−7^	4.24 e^−5^
Wnt signaling pathway	151	17	1.23 e^−6^	4.58 e^−5^
Basal cell carcinoma	55	10	2.59 e^−6^	8.03 e^−5^
Hedgehog signaling pathway	56	9	2.33 e^−5^	5.99 e^−4^
Melanogenesis	101	12	2.58 e^−5^	5.99 e^−4^
Cell adhesion molecules (CAMs)	133	13	9.51 e^−5^	1.97 e^−3^
Endocytosis	181	15	1.86 e^−4^	3.45 e^−3^
Axon guidance	129	12	2.78 e^−4^	4.7 e^−3^
Small cell lung cancer	84	9	5.65 e^−4^	8.19 e^−3^
MAPK signaling pathway	267	18	5.73 e^−4^	8.19 e^−3^
O‐Glycan biosynthesis	30	5	1.34 e^−3^	1.68 e^−2^
Phenylalanine metabolism	18	4	1.35 e^−3^	1.68 e^−2^
Intestinal immune network for IgA production	48	6	2.11 e^−3^	2.42 e^−2^
Glioma	65	7	2.21 e^−3^	2.42 e^−2^
Melanoma	71	7	3.66 e^−3^	3.78 e^−2^
Dorso‐ventral axis formation	24	4	4.11 e^−3^	4.03 e^−2^
Calcium signaling pathway	178	12	4.47 e^−3^	4.16 e^−2^
Maturity onset diabetes of the young	25	4	4.79 e^−3^	4.24 e^−2^

### 3.5 Association with mortality

**FIGURE 5:**
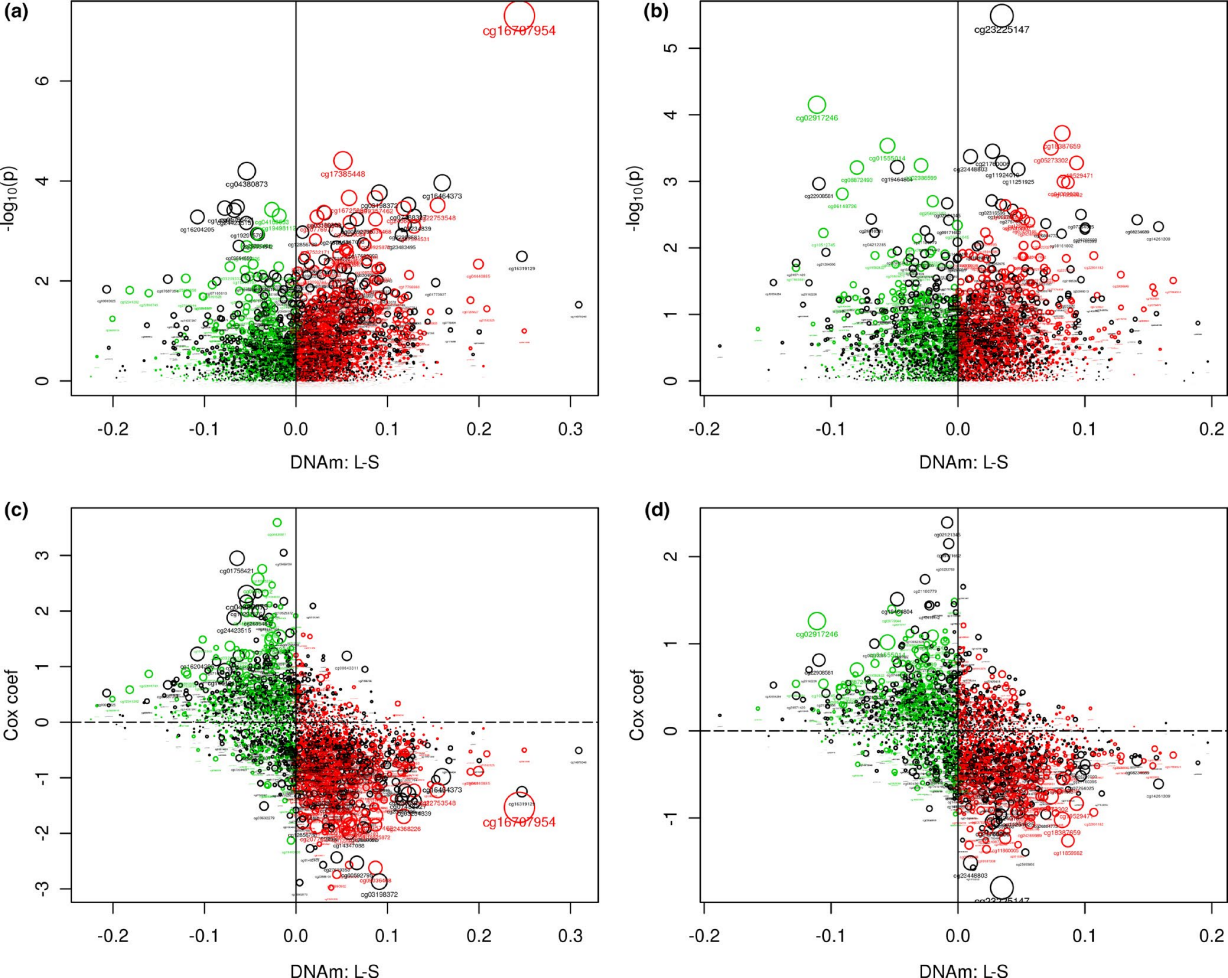
Association with mortality by discovery CpGs in old LSADT twins shown by plotting negative log of the p value (base 10) for each CpG site (y‐axis) against intra‐pair L‐S difference in DNAm for male (a) and female (b) twins and by plotting Cox regression coefficient of each CpG site against intra‐pair L‐S difference in DNAm for male (c) and female (d) twins. The colored dots are CpGs overlapping CpGs between male and female samples with L‐S>0 (red dots) or L‐S<0 (green dots)

For the 3089 discovery CpGs, 2906 CpGs were matched to the LSADT methylation data. We fitted Cox regression models to the 2906 CpGs adjusting for age at blood sampling and intra‐pair correlation as described in the Materials and Methods section. In male twins, 264 CpGs showed significant association with risk of death with *p *< 0.05 (Table S3). One CpG even meets significance after Bonferroni correction (*p* < 1.72e‐05). A binomial test with a type I error rate of 0.05 as the hypothesized probability of success showed that the correlation with mortality by the discovery CpGs is extremely unlikely by chance (*p* < 2.2e‐16). In female samples, 175 CpGs were correlated with mortality with *p*<0.05, 1 CpG with a *p* value below Bonferroni significance (*p* = 3.24e‐06) (Table S3). Though the discovery CpGs are from male MADT samples, a binomial test still showed a *p* value of 0.013 indicating a significant, non‐random implication of these CpGs in female survival. In Figure S4, the coefficients for DNAm in the Cox model are plotted against their *p* values. We can see that there are slightly more CpGs with negative coefficients (increased DMAm reduces risk of death or decreased DNAm increases risk of death) than CpGs with positive coefficients (1783 negative, 1123 positive in males; 1605 negative, 1301 positive in females). The beneficial effect of increased DNAm of the discovery CpGs on survival is further supported by plotting their Cox p values against the mean intra‐pair DNAm difference between longer and shorter survivors (L‐S) (Figure 5a, 5b). For most of the discovery CpGs, the longer‐lived twins tend to have higher DNAm than their shorter‐lived co‐twins, or shorter‐lived twins tend to have lower DNAm than their longer‐lived co‐twins (CpGs with L‐S > 0, 1594 and 1660 CpGs in the right panels of Figure 5a and 5b). The left panels of Figure 5a and 5b show CpGs with lower DNAm in longer survivor or higher DNAm in shorter survivor within each pair (CpGs with L‐S < 0, 1312 and 1246 CpGs in the left panels of Figure 5a and 5b, respectively). The patterns in Figure 5a and 5b are weak but it is consistent for both males and females, with a slightly higher proportion of CpGs with L‐S > 0 that favor survival in female (57.12%) than in male (54.85%) twins (*p *= 0.086), although the discovery CpGs were found in male twins. The red (L‐S > 0 CpGs) and green (L‐S < 0 CpGs) dots are CpGs showing consistent signs for L‐S in the two sexes, accounting for 51% of the 2906 CpGs. In Figure 5, we also plot L‐S in male (5c) and female (5d) twins against Cox regression coefficient in survival analysis for each of the 2906 CpGs. It is evident that CpGs with higher DNAm in longer survivors or with lower DNAm in shorter survivors mostly have negative Cox regression coefficients while CpGs with higher DNAm in shorter survivors or with lower DNAm in longer survivors have positive Cox regression coefficients in both male and female samples.

### 3.6 Verification of mortality association

The MADT twins have a maximum age of 80 and a mean age of 66 years. A total of 59 twins are deceased (40 males, 19 females) with a median age of 78 (range: 63–88) (Table 1). Although the count of events is small, we tried to use the data to test if the L‐S > 0 CpGs and L‐S < 0 CpGs identified in LSADT twins are predictive of mortality in an independent cohort. To do that, we first took the 1594 CpGs with L‐S > 0 and 1312 CpGs with L‐S < 0 in male LSADT twins and calculated mean of each group for male and for female MADT twins separately, considering L‐S > 0 and L‐S < 0 CpGs have opposite effects on survival and then fitted Cox regression models for each sex with the calculated means as covariates. In the fitted Cox model applied to male MADT twins, the mean of L‐S > 0 CpGs significantly reduced the risk of death (*Z* = −3.17, *p* = 1.51e‐03) while the mean of L‐S < 0 CpGs significantly increased the risk (*Z* = 3.54, *p* = 4.04e‐04). Surprisingly, in female MADT twins, even though there are only 19 registered deaths and the L‐S CpGs are defined by male twins, the applied Cox model still estimated a significantly beneficial effect for the mean of L‐S > 0 CpGs (*Z* = −2.49, *p* = 1.27e‐02) and a significantly harmful effect for the mean of L‐S < 0 CpGs (*Z* = 2.68, *p* = 7.33e‐03).

## 4 DISCUSSION

We have conducted an EWAS on age‐related intra‐pair DNAm discordance in MZ twins to identify CpGs with high variability in their DNAm levels with increasing age in male and female samples separately and assessed their impact on risk of death. The advantages in using twins for EWAS on DNAm variability are multifold: (1) individual difference in level of DNAm (variability) can be matched for chronological age for assessment across ages; (2) individual genetic make‐ups are perfectly matched for effectively controlling potential genetic influences on DNAm level so that the identified age‐related DNAm variability patterns are independent of individual genetic variations and are purely associated with environmental factors or stochastic events. This is highly relevant with respect to mortality as it has been estimated that genetic variation only account for around 25% of human lifespan variation (Herskind et al., 1996); (3) with identical twin pairs, covariates such as anthropometric measurements can be canceled by studying intra‐pair difference on methylation. As a result, the use of MZ twin pairs helped to enrich the power of this study.

The increased intra‐pair DNAm variability with age was already reported by Fraga et al., (2005) and Boks et al., (2009) but with no sex differences analyzed, perhaps due to the very small sample sizes studied. Using unrelated individuals, Slieker et al., (2016) reported 6366 CpGs displaying age‐related methylation variability in a sex combined analysis. A hypergeometric test showed that there is an extremely significant overlap (256 CpGs) by our 3084 discovery CpGs with their reported CpGs (*p* < 1.29e‐111). Most importantly, the striking difference in the number of CpGs demonstrating age‐related DNAm variability between males and females in this study (Figure 1) suggests that the female epigenome could be more stable and less vulnerable during the aging process which may help to maintain genomic stability and expand female healthspan as well as lifespan. The fact that the observed sex difference was replicable in an independent sample (Figure 3) suggests that the age‐related DNAm variability is stable across samples, while the high replication rate of 37% in an independent twin cohort indicates that specific CpGs might be more responsive to environmental stimuli in males than in females. In Figure 1, nearly all significant CpGs (FDR < 0.05) are characterized by increased intra‐pair DNAm discordance or epigenetic variability which could reflect the accumulative effects of environmental exposure during the life course which is, as shown by our results, more pronounced in males than in females perhaps due to biological mechanisms.

In the discovery EWAS for female twins (Table S1), no CpG reached genome‐wide significance of FDR < 0.05 although the *p* value of the top CpG (cg14239986) is 4.69e‐07. In the discovery EWAS for male twins, however, the genome‐wide significance of FDR < 0.05 corresponds to a *p* value of 5.25e‐04. This difference in *p* values corresponding to genome‐wide significance as defined by FDR < 0.05 is due to the fact that the FDR is calculated on all test statistics for male and female samples separately, that is, the *p* value corresponding to a FDR is sample‐specific and not comparable across samples (Higdon et al., 2008). Nevertheless, it is important to note that, similar to the discovery EWAS for male twins, the top CpGs from the discovery EWAS for female twins (Table S1) also display a predominant pattern of increased variability with age although not genome‐wide significant. Among the top 18 CpGs with *p* < 1e‐04 (Table S1), 5 CpGs (cg07596529, cg09529138, cg00846166, cg22112832, cg09578353) show same direction of age‐related increase in variability with *p *< 0.05 in male discovery twins (accounting for 28%). The pattern is also displayed in Figure 1b suggesting that the age‐related epigenetic instability is also observed in females but not as striking as in males (Figure 1a).

In Table 2, the genes linked to the top significant CpGs (*p* < 1e‐07) are all reported to associate with aging‐related pathologies in previous publications. *ELFN1* is a synaptic adhesion protein important in formation and maintenance of synapses and regulation of synaptic plasticity. Alterations in synaptic adhesion play a key role in the disruption of neuronal networks in Alzheimer's disease (Leshchyns'ka & Sytnyk, 2016). Differential methylation in the intronic region of the *C1QL4* gene has been found in a EWAS of coronary artery disease patients (Sharma et al., 2014). *FAM19A1* mRNA expression is restricted to the central nervous system, and the expression level of the gene correlates with brain development and neurogenesis (Zheng et al., 2018). Genetic variation of *FAM19A1* has been found to associate with human longevity in a large scale genome‐wide association study (Zeng et al., 2018). Decreased expression of *SULF2* was found in Alzheimer's disease patients indicating its implication in regulating neuronal signaling (Roberts et al., 2017).

Biological pathway analysis by GSEA (Table 3) showed enriched KEGG pathways involved in cancers, multiple signaling pathways and metabolism, underlying major aging‐associated diseases and traits. Interestingly, the significantly enriched biological pathways (Table 3) contain a number of important evolutionarily well‐conserved signal transduction pathways of aging, that is, the WNT (Wnt) and Hedgehog (Hh) signaling pathways. Effective interactions within key signal transduction networks determine success in embryonic organogenesis, and postnatal tissue repair throughout developmental stages and aging (Carlson et al., 2008). Imbalances within these pathways during aging could be speculated to be responsible for age‐related degenerative diseases and phenotypes. Both the Wnt and Hh pathways have shown implications in a variety of cancers (Taipale & Beachy, 2001), and it is possible that aberrant Hh pathway activation manifests cancer phenotypes by directly altering normal Wnt signaling through Wnt‐Hh cross‐talk (Carlson et al., 2008).

By performing survival analysis on a cohort of elderly twins (LSADT cohort), we were able to show the extensive implication of the identified age‐related variability CpGs in individual's risk of death in male twins. Interestingly, the variability CpGs detected in males also showed non‐random effects on female survival both in survival analysis by Cox regression and in intra‐pair differential methylation comparison between longer‐ and shorter‐lived twins. In Figure 5, it is clearly shown that the age‐related variability CpGs are grouped into those with negative Cox coefficients and L‐S > 0 and those with positive Cox coefficients and L‐S < 0 in both sexes with the former accounting for most of the variable CpGs. The significant involvement of the variable CpGs in mortality is further confirmed by survival analysis on MADT twins with significant coefficients for variables defined as the mean DNAm of L‐S > 0 and mean DNAm of L‐S<0 CpGs. Again the significant association is observed in both male and female MADT twins, even though with limited death counts in the MADT cohort.

It is hypothesized that the DNA methylome, evolved to increase stability of the differentiated state in somatic cells may have helped to increase longevity (Mendelsohn & Larrick, 2013). Considering the fact that a very large proportion of CpG sites in the genome have methylated cytosines (75–85%) (Kojima et al., 2018), the CpGs with L‐S>0, which account for most of the significantly variable CpGs, could indicate that decreased DNAm or loss of control over gene activity is harmful to survival. The observed significant involvement in mortality by the highly variable CpGs in males could potentially help to explain the female survival advantage. Given the limited genetic contribution to human lifespan (Herskind et al., 1996; Ruby et al., 2018), studying the environment‐mediated epigenetic instability in aging may hold the key to promoting healthy aging and extending human life span.

## 5 CODE AVAILABILITY

All R codes are used for calculation and data analysis are available upon contact with the corresponding author at qtan@health.sdu.dk.

## CONFLICT OF INTERESTS

None declared.

## AUTHOR CONTRIBUTIONS

KC, QT, and SL conceived the study. QT performed statistical and bioinformatics analysis. SL, JM, and QT interpreted the biological findings. MS, MN, and JM prepared data, quality control, and functional annotation analysis. KC is in charge of coordinating and organizing the whole study.

## Supporting information

Fig S1

Fig S2

Fig S3

Fig S4

Table S1

Table S2

Table S3

## Data Availability

According to current Danish and EU legislations, transfer and sharing of individual‐level data require prior approval from the Danish Data Protection Agency. Our present local data protection rules do not allow individual‐level data to be shared in public databases. For these reasons, the raw data cannot be deposited in a public database. However, we welcome any enquiries regarding collaboration and individual requests for data sharing. Requests can be directed to Qihua Tan at qtan@health.sdu.dk.
