# Supplementary figures and images for: Age patterns of intra‐pair DNA methylation discordance in twins: Sex difference in epigenomic instability and implication on survival
Source: Aging Cell. 2021 Aug 24;20(9):e13460. doi: 10.1111/acel.13460 (PMC8441297; doi:10.1111/acel.13460)

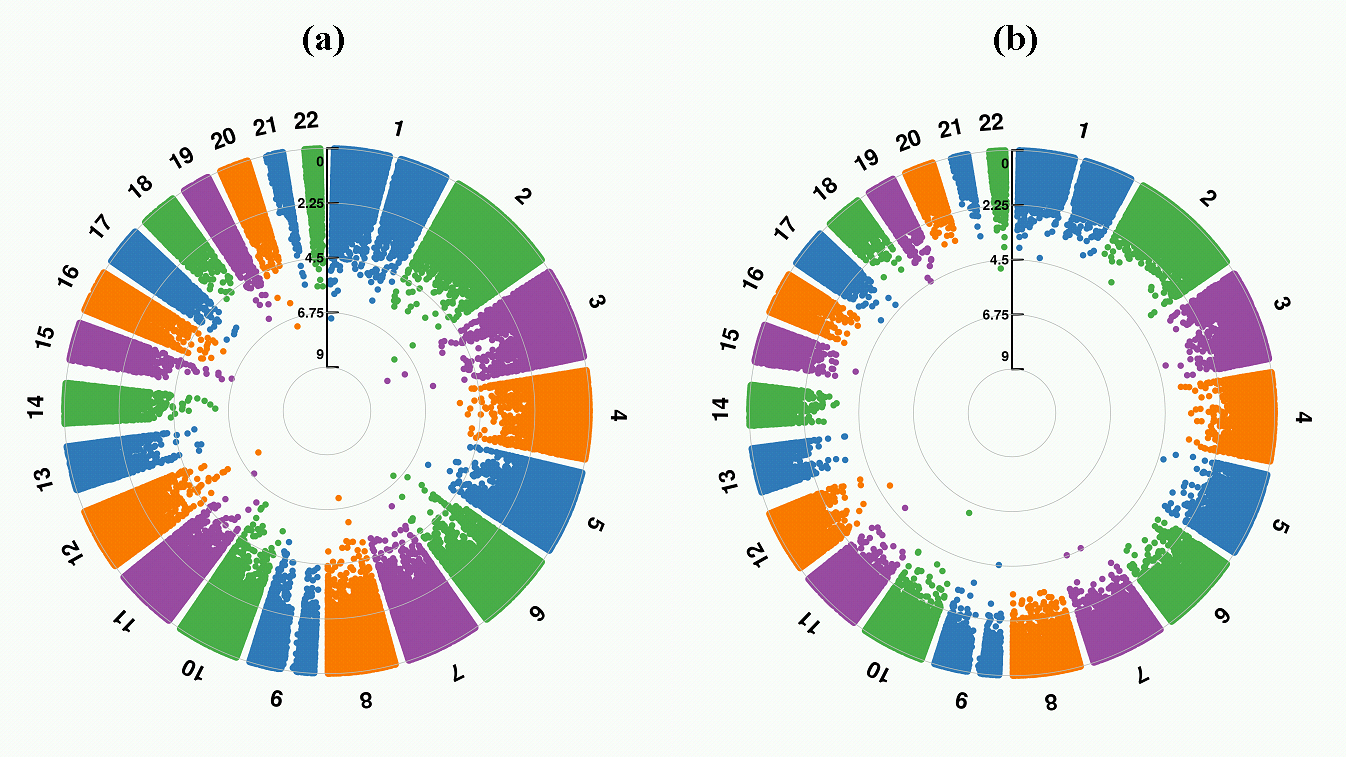

Supplement: Supplementary file 1 — Fig S1 [file ACEL-20-e13460-s002.png]

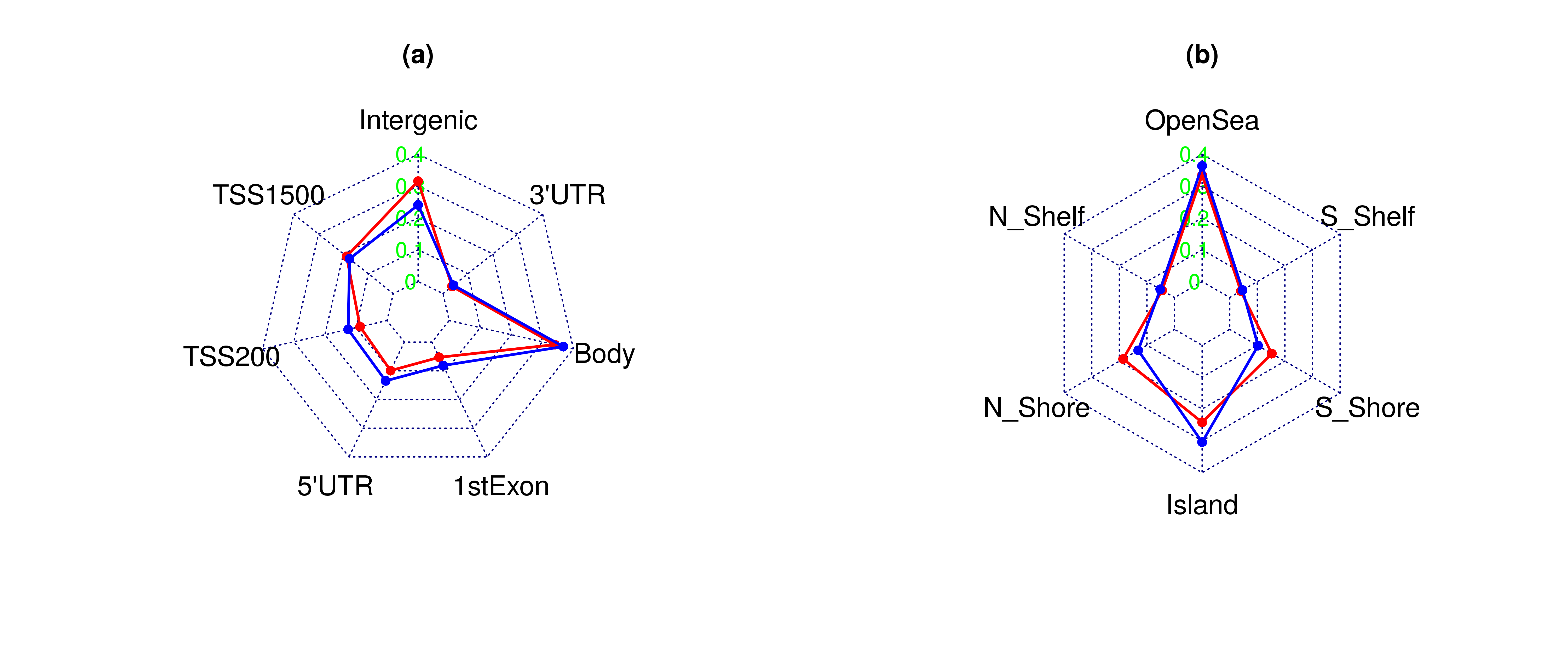

Supplement: Supplementary file 2 — Fig S2 [file ACEL-20-e13460-s006.png]

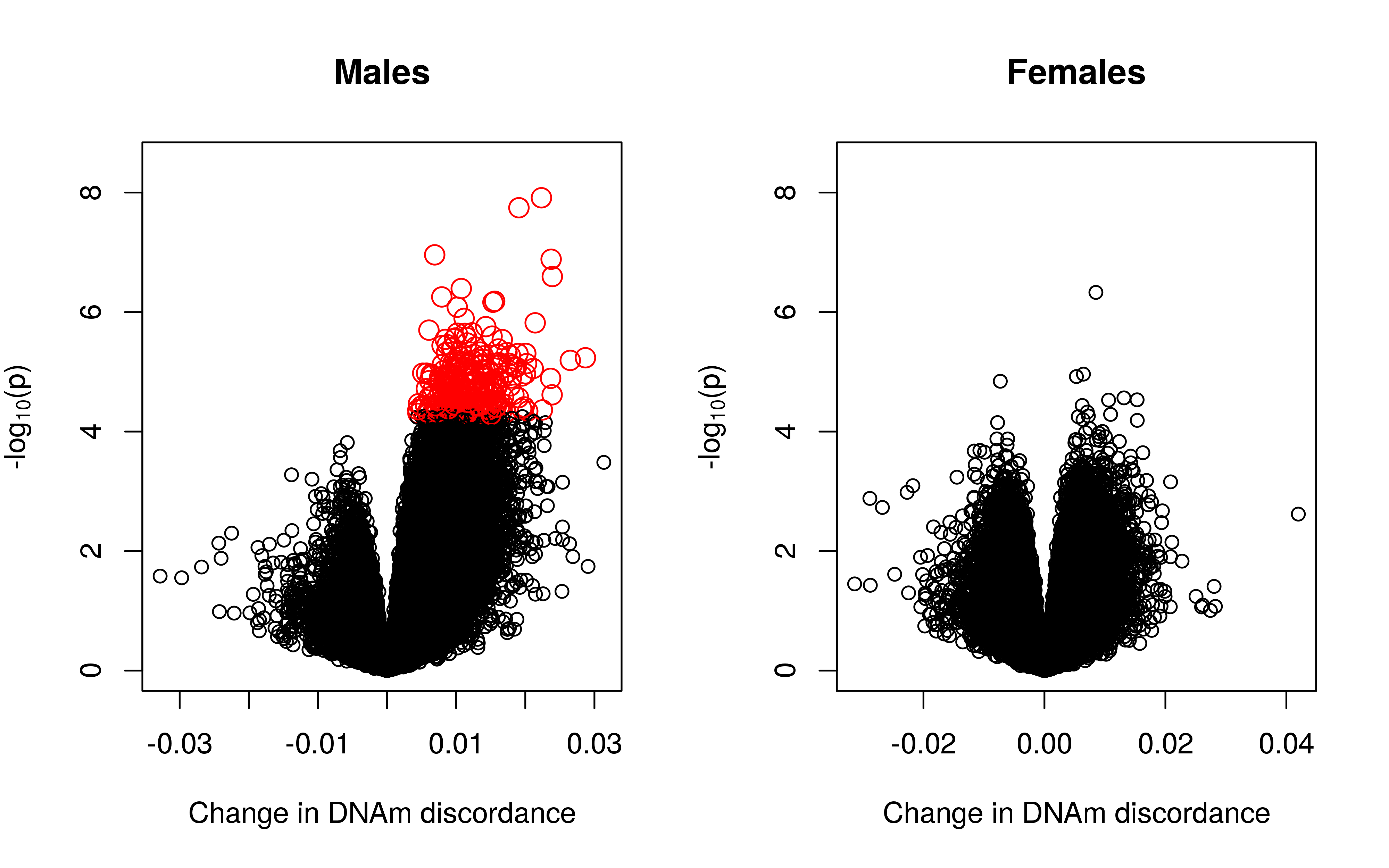

Supplement: Supplementary file 3 — Fig S3 [file ACEL-20-e13460-s001.png]

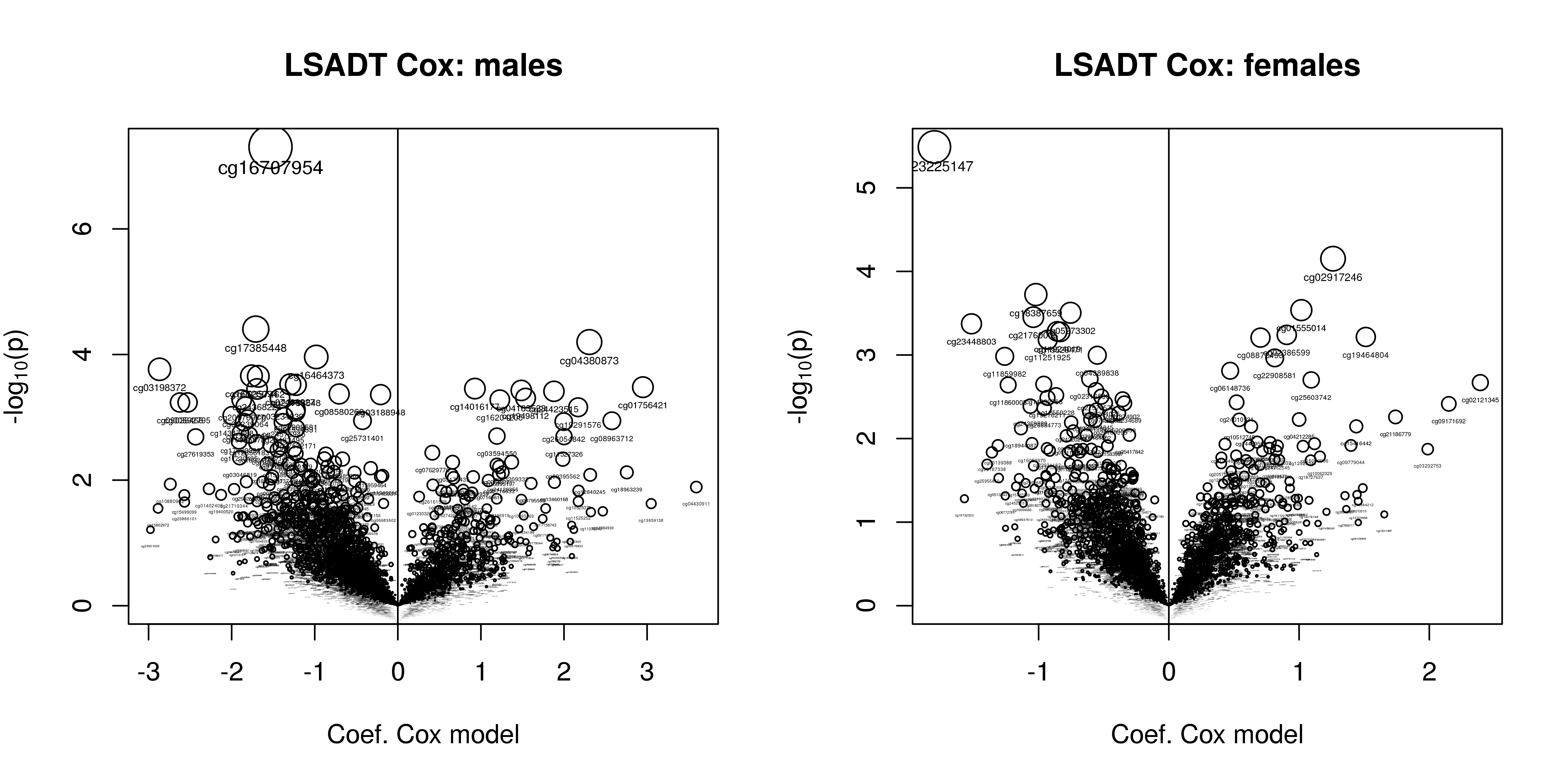

Supplement: Supplementary file 4 — Fig S4 [file ACEL-20-e13460-s007.png]
